# AGE DIFFERENCES IN THE EFFECTS OF PERIODONTAL TREATMENTS ON ORAL AND METABOLIC HEALTH IN PEOPLE WITH DIABETES

**DOI:** 10.1093/geroni/igad104.1323

**Published:** 2023-12-21

**Authors:** Zheng Zhu, Xiang Qi, Yaguang Zheng, Yaolin Pei, Bei Wu

**Affiliations:** New York University, New York City, New York, United States; New York University, New York City, New York, United States; New York University, New York City, New York, United States; New York University, New York City, New York, United States; New York University, New York City, New York, United States

## Abstract

**Background:**

This systematic review aims to explore the age differences in the effect of multi-component periodontal treatments on oral and metabolic indicators among individuals with periodontitis and diabetes.

**Methods:**

Six databases were searched from database inception to August 2022. Trials reporting the effects of multi-component periodontal treatments on oral and metabolic indicators among participants aged 18 and above with periodontitis and diabetes were included. We used bivariate and multivariate meta-regression models to examine the association between age and treatment effect size. The primary outcomes were changes in probing depth (PD), clinical attachment level (CAL), and hemoglobin A1c (HbA1c).

**Results:**

A total of 115 trials (119 articles) met inclusion criteria. We found that multi-component periodontal treatment significantly reduce PD (g=0.929, 95% CI [0.689-1.169], I2=94.1%), CAL (g=0.879, 95% CI [0.669-1.089], I2=92.1%), and HbA1c (g=0.603, 95% CI [0.443-0.763], I2=87.5%). A significant decreasing trend was observed in the effect size for PD (P for trend = 0.020) and CAL (P for trend = 0.028) as age increases. Results from multivariate meta-regression showed that age was associated with smaller effects of PD (β [standard error] = -0.123 [0.041], P=0.004) and CAL (-0.159 [0.055], P=0.006). Compared to their younger counterparts, the effect size for HbA1c was smaller among participants aged 55 and older (-0.792 [0.322], P=0.017).

**Conclusions:**

The effect of multi-component periodontal treatments on PD, CAL, and HbA1c may be more effective among younger populations. Our study suggests that tailored interventions are needed to improve oral health and diabetes management for individuals with periodontitis and diabetes.

